# Sutureless technique for left pulmonary vein occlusion with persistent left superior vena cava: Case report

**DOI:** 10.1016/j.xjtc.2024.06.013

**Published:** 2024-06-28

**Authors:** Kazuki Tamura, Joji Hoshino, Masahiko Ezure, Yutaka Hasegawa, Yasuyuki Yamada, Shuichi Okada, Hiroyuki Morishita, Masahiro Seki, Takashi Soda

**Affiliations:** Division of Cardiovascular Surgery, Gunma Prefectural Cardiovascular Center, Maebashi, Japan

**Figure:**
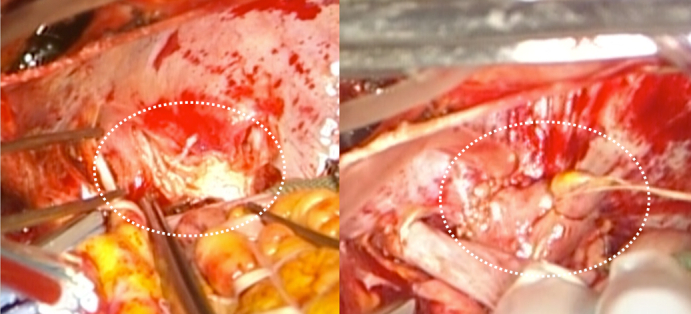
Sutureless technique for the occlusive left PV with left SVC.

**Central Message**

We performed a surgical repair using the sutureless technique with complete occlusion of the left PV, complicated by persistent left SVC, and achieved very good results.

Pulmonary vein stenosis (PVS) after catheter ablation (CA) is a relatively uncommon complication, and surgical repair is an option in severe stenosis or occlusion cases. We report a rare case of surgical repair using the sutureless technique for complete occlusion of the left pulmonary vein (PV) after CA complicated with persistent left superior vena cava (PLSVC).

## Case

**Figure 1:**
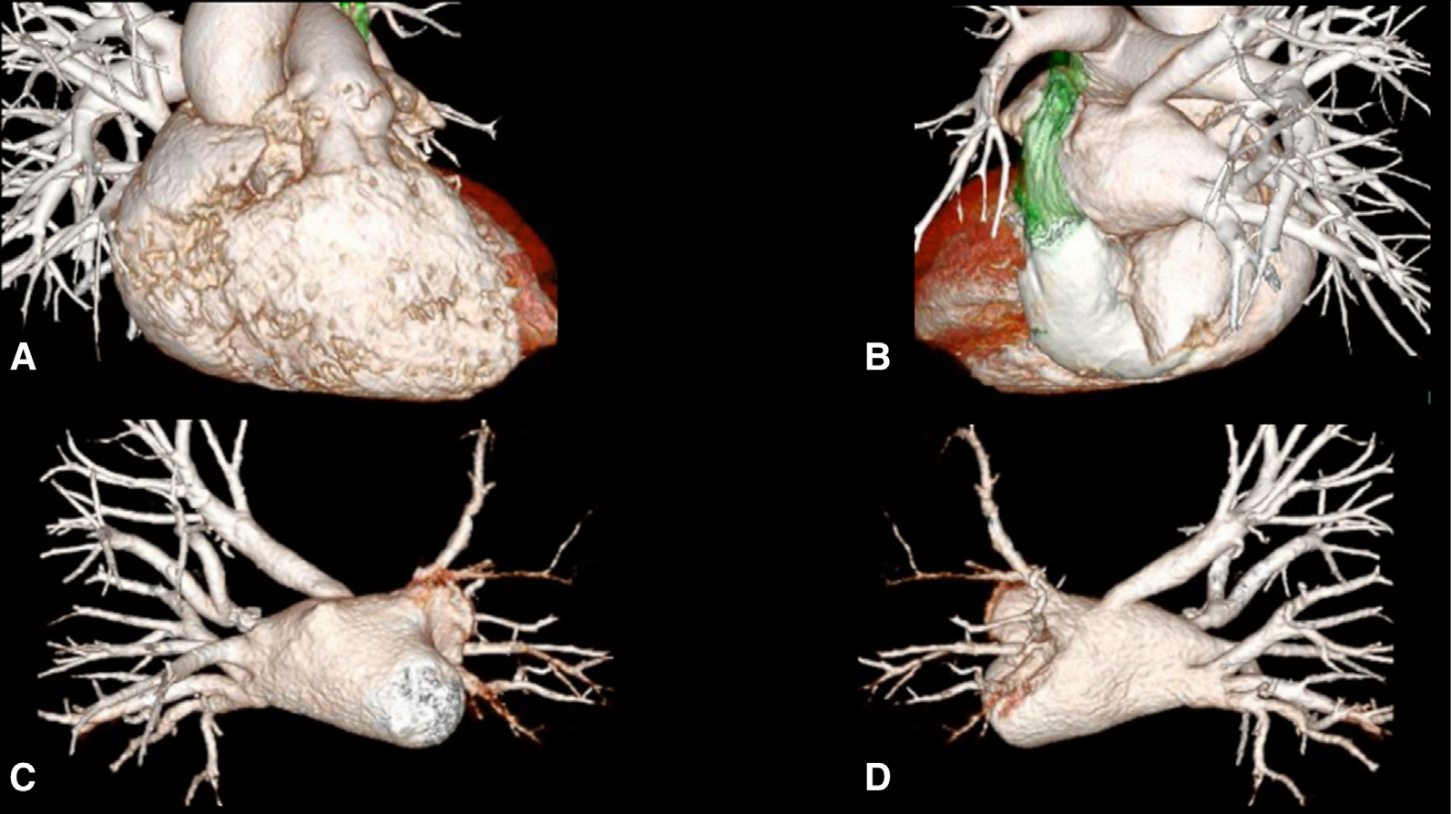
A and B, Preoperative computed tomography revealed complete occlusion of the PVs. The *green* structure represents the PLSVC. C and D, Postoperative computed tomography: The PV branches were patent.

The patient is a 51-year-old woman complicated with PLSVC. One year ago, she was diagnosed with atrial fibrillation (AF), right ventricular enlargement, and severe tricuspid regurgitation. Nine months ago, the patient underwent CA (PV, superior vena cava [SVC], and cavo-tricuspid isthmus isolation). There was no AF recurrence, and the patient was monitored at another hospital. Eight months after the CA, the patient presented with hemoptysis, and computed tomography imaging revealed left PV obstruction (Figure 1, *A* and *B*). Echocardiography revealed severe tricuspid regurgitation with an ejection fraction of 60%. No abnormalities were detected in the other valves. Right heart catheterization showed no difference in pulmonary wedge pressure between the right and left pulmonary arteries (pulmonary capillary wedge pressure: right/left = 15/16 mm Hg), with a mean pulmonary artery pressure of 23 mm Hg in the right pulmonary artery, although measuring in the left posed challenges. The maximal right ventricular pressure was found to be elevated at 31 mm Hg and ventilation-perfusion scintigraphy revealed a left/right mismatch ratio of 9.2:90.8. Respiratory symptoms worsened with hemoptysis, prompting the decision for surgical treatment because catheterization was not feasible. Surgery commenced with a median sternotomy under general anesthesia. Initially, the left SVC was identified outside the pericardium. Cardiopulmonary bypass was then established via ascending aortic cannulation and venous drainage from both the SVCs, as well as the inferior vena cava. Intraoperative observation revealed that the PLSVC coursed posteriorly to the left atrial appendage (LAA) and anteriorly to the PV, with mild adherence to the left atrial (LA) wall. The PLSVC was carefully detached from the LA wall, and the left upper and lower PVs were incised. Both of them were completely obstructed with fibrotic intimal thickening. The LAA was guided through the dorsal aspect of the dissected PLSVC to restore the left PVs and LAA to their normal anatomic position. An LAA flap was created by extending the incision from the LA wall to the LAA. The PVs were covered by the LA and LAA flap, which were sutured to the pericardium surrounding the PVs using a 5-0 monofilament running suture under hypothermic circulatory arrest. The tricuspid valve exhibited an enlarged valve annulus, and tricuspid annuloplasty was performed. The operation time was 356 minutes, extracorporeal circulation time was 230 minutes, and circulatory arrest time was 24 minutes. The postoperative course was uneventful. A contrast-enhanced computed tomography scan revealed patency of both PVs (Figure 1, *C* and *D*), and ventilation-perfusion scintigraphy showed significant improvement with a left-to-right ratio of 22.4:77.6. The patient was discharged after 33 days with significant improvement in symptoms. We have followed the patient for 1 year, and she remains free of symptoms (Video 1).

## Discussion

In general, PLSVC occurs at a frequency of 0.3% to 0.5% and is associated with 3% to 10% of congenital heart disease.^1^ Although there have been reports of surgical repair for congenital PVS complicated by PLSVC, such cases in adults are rare.^2^ PVS has various causes, including congenital, neoplastic, and idiopathic origins. However, the most common cause in recent years has been idiopathic, resulting from CA of the PVs for AF.^2^ Its frequency has decreased to approximately 2% from an average of 6.3% between 1999 and 2004. However, this may be underestimated because of short postoperative follow-up periods, typically approximately 3 months, and asymptomatic patients are not always imaged.^2^ The cause of PVS after CA is thought to involve thrombus formation, epithelialization, proliferative neointimal hyperplasia, and angiogenesis induced by radiofrequency ablation.^2^^,^^3^ Clinical symptoms typically manifest 3 to 6 months after CA and include chest pain, dyspnea, cough, hemoptysis, recurrent pulmonary infection, and pulmonary hypertension. In this case, hemoptysis manifested 8 months postoperatively, which is a relatively late onset, and the PV was completely occluded, potentially delaying the diagnosis. Surgical repair for PVS with the sutureless technique reduces the risk of anastomotic stenosis and thrombus formation by avoiding direct anastomosis.^4^ This surgical technique is particularly useful for left PVS, using the LAA as a flap. In this case, despite the challenge posed by the PLSVC positioned between PVs and LAA, we successfully performed the sutureless technique by detaching the PLSVC from the LA wall and relocating the LAA to its dorsal side. Anatomic repair of the PLSVC to the SVC was not performed because the PLSVC itself does not cause hemodynamic abnormalities and is typically asymptomatic. In this case, anatomic repair was deemed unnecessary because the PLSVC was connected to the right atrium and there was no structural compression of the PV. Furthermore, repairing PLSVC requires careful consideration due to reported pulmonary embolism from thrombus formation at the anastomotic site.^5^ This case serves as a valuable example demonstrating that PV occlusion can be successfully repaired using the sutureless technique, even in adult patients complicated by PLSVC.

## Conclusions

Effective PV patency can be achieved by using the sutureless technique even in patients with PLSVC and for PV occlusion after CA in adult patients. Careful outpatient follow-up is necessary, because the risk of restenosis in cases of PLSVC complications is unknown.

## Conflict of Interest Statement

The authors reported no conflicts of interest.

The *Journal* policy requires editors and reviewers to disclose conflicts of interest and to decline handling or reviewing manuscripts for which they may have a conflict of interest. The editors and reviewers of this article have no conflicts of interest.

## Supplementary Data

Video 1 The PLSVC coursed posteriorly to the LAA and anteriorly to the PV. We then performed a sutureless technique using the LAA as a flap under hypothermic circulatory arrest. Video available at: https://www.jtcvs.org/article/S2666-2507(24)00262-1/fulltext.
