# Supplementary figures and images for: Sutureless technique for left pulmonary vein occlusion with persistent left superior vena cava: Case report
Source: JTCVS Tech. 2024 Jun 28;27:119–21. doi: 10.1016/j.xjtc.2024.06.013 (PMC11518861; doi:10.1016/j.xjtc.2024.06.013)

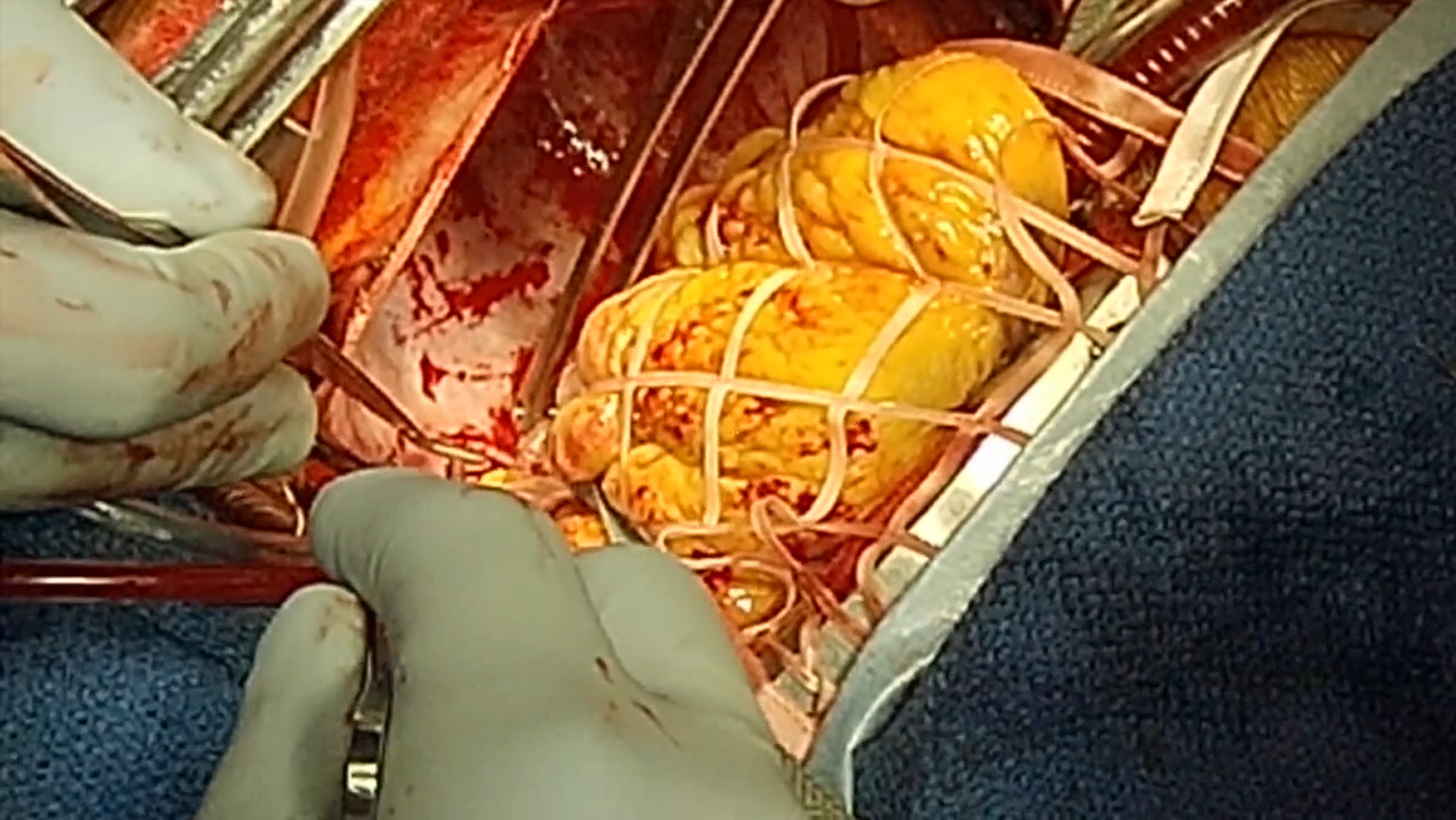

Supplement: Video 1 — The PLSVC coursed posteriorly to the LAA and anteriorly to the PV. We then performed a sutureless technique using the LAA as a flap under hypothermic circulatory arrest. Video available at: https://www.jtcvs.org/article/S2666-2507(24)00262-1/fulltext. [file fx2.jpg]
